# Dr. Jacob A. Cook

**Published:** 1853-10

**Authors:** 


### DR. JACOB A. COOK.

We would be pleased to learn the location of this gentleman. He has been in the profession for about eight years, and was, we think, at one time, a resident of Bourbon Co., Ky.

